# Preparation and Characterization of Cu-Mn-Ce@γ-Al_2_O_3_ to Catalyze Ozonation in Coal Chemical Wastewater-Biotreated Effluent

**DOI:** 10.3390/ijerph16081439

**Published:** 2019-04-23

**Authors:** Yue Teng, Ke Yao, Wenbin Song, Yongjun Sun, Haoliang Liu, Zhiying Liu, Yanhua Xu

**Affiliations:** 1 College of Environmental Science and Engineering, Nanjing Tech University, Nanjing 211800, China; sunadnmoon19960203@163.com (Y.T.); yaoke214421@163.com (K.Y.); swb19960311@163.com (W.S.); 2 College of Urban Construction, Nanjing Tech University, Nanjing 211800, China; sunyongjun@njtech.edu.cn; 3 NJTECH Environment Technology Co., Ltd., Nanjing 210000, China; liuhaoliang@163.com

**Keywords:** catalyst, catalytic ozonation, coal chemical wastewater, advanced treatment

## Abstract

Cu-Mn-Ce@γ-Al_2_O_3_ was prepared by incipient wetness impregnation and used to catalyze ozonation in a coal chemical wastewater-biotreated effluent. The preparation factors that considerably affected the catalytic performance of Cu-Mn-Ce@γ-Al_2_O_3_, specifically metal oxide loading percentage, calcination temperature, and calcination time, were examined. The catalyst was characterized by scanning electron microscopy, energy dispersive spectrometry, X-ray diffraction, and Brunauer-Emmett-Teller analysis. The optimal catalytic ozonation operating parameters, such as ozone dosage, catalyst dosage, pH, and reaction time, were also investigated. Results showed that an optimized catalyst consisted of 17.0% CuO, 3.0% MnO_2_, and 2.0% CeO_2_ (wt.%). The optimal calcination temperature and calcination time were 600 °C and 5 h. The optimal catalytic ozonation operating parameters, including ozone dosage, catalyst dosage, pH, and reaction time, were 7, 80.0 mg/L, 20.0 mg/L, 7 and 50 min, respectively. The COD removal of biotreated effluent increased to 61% under these optimal operating conditions. Meanwhile, ozonation alone resulted in only 20% removal. This work proposes the use of easily available Cu-Mn-Ce@γ-Al_2_O_3_ catalyst and might drive the advancement of catalytic ozonation for chemical wastewater purification.

## 1. Introduction

Coal chemical wastewater is one kind of organic wastewater which is difficult to treat. Even after biological treatments, there are still residual recalcitrant compounds due to the presence of biologically inhibitory organic substances. Coal chemical wastewater-biotreated effluent contains a large amount of toxic and refractory organic pollutants (TROPs), such as phenolic compounds, polycyclic aromatic hydrocarbons, nitrogenous heterocyclic compounds (NHCs), long-chain hydrocarbons, and ammonia [1]. TROPs inhibit the growth of microorganisms by influencing biodegradation; once released into the environment, TROPs present significant threats to the aquatic environment and human health [2] because of their toxicity, carcinogenicity, and mutagenicity [3]. In recent years, this wastewater treatment has become a bottleneck for the development of the coal chemical industry in China and has encouraged researchers to develop advanced methods.

In recent years, the advanced treatment methods of coal chemical wastewater mainly include coagulation, membrane filtration, adsorption, and advanced oxidation processes (AOPs). For example, Ma et al. treated the coal gasification wastewater-biotreated effluent with a combination of a submerged ultrafiltration membrane and reverse osmosis membrane to remove more than 80% of COD and ammonia nitrogen in wastewater [4]. These methods, such as membrane filtration and adsorption, tend to be effective across the board at removing the TROPs. However, they can only remove the pollutant and not transform it, which generates a hazardous waste stream that has to be dealt with. Among AOPs, ozonation is a better method for advanced treatment of coal chemical wastewater-biotreated effluent.

Ozonation can be an effective technology for the removal of TROPs in coal chemical wastewater-biotreated effluent. Ozone (redox potential of 2.07 V), as a powerful oxidant, has received much attention for its high oxidation capacity [5], and it can selectively oxidize unsaturated double bonds and aromatic structures. Ozone attacks organic compounds through the following mechanisms [6]: (1) direct ozonation by ozone molecules, and (2) indirect free-radical mechanism, involving highly oxidative hydroxyl radical. Ozonation has the limitations of short half-life, and it requires continuous ozone generation, which increases the energy consumption and operating cost of the process [7]. Moreover, ozonation alone cannot completely degrade organic compounds, and it sometimes produce toxic intermediates [8]. Therefore, ozone generation and use must be optimized so that the cost of effluent treatment can be greatly reduced.

Catalyst addition in ozonation, called catalytic ozonation, is known as an effective advanced oxidation process (AOP) that removes TROPs from coal chemical wastewater-biotreated effluent. The catalyst in ozonation promotes decomposition of ozone on its surface to produce hydroxyl radicals (·OH) (redox potential of 2.80 V). Single ozonation cannot completely oxidize recalcitrant organic compounds, but they can be successfully oxidized via catalytic ozonation [9]. Catalytic ozonation is classified into homogeneous and heterogeneous types on the basis of the type of catalyst used in the process. In homogeneous catalytic ozonation, ozone is decomposed with transition metal ions. In heterogeneous catalytic ozonation, solid catalysts are used. However, the use of transition metal ions in homogenous systems is restricted because they are mostly harmful to the environment and must be separated from a treated effluent. The separation of transition metal ions is challenging because of their extremely low concentrations in water. These limitations are overcome by using solid catalysts, which are easy to segregate and can maintain catalytic activity for a long time [10]. Therefore, heterogeneous catalytic ozonation may be more effective than conventional ozonation in eliminating TROPs from the coal chemical wastewater-biotreated effluent [11]. In heterogeneous catalytic ozonation, a solid catalyst is utilized to accelerate ozone decomposition to generate hydroxyl radicals, which can non-selectively oxidize or mineralize organic contaminants. Furthermore, solid catalysts enhance organic pollutant degradation by adsorbing them on its surface to accelerate the reaction between pollutant and ozone. A variety of metal oxides, either loaded or unloaded, are widely investigated as catalysts in heterogeneous catalytic ozonation [12]. The compound γ-Al_2_O_3_, or transition metal oxide-loaded γ-Al_2_O_3_, has been extensively studied as a catalyst owing to its structural stability, environment friendliness, low cost, and high industrial feasibility [13]. The surface basic sites, e.g., hydroxyl (-OH) groups of γ-Al_2_O_3_, contribute to the generation of ·OH, and the porous structure promotes the adsorption of TROPs [14,15]. Amir et al. investigated the mechanisms of ozonation in the presence of γ-Al_2_O_3_. They stated that alumina greatly promote the formation of reactive oxygen species (ROS) in aqueous solutions relative to ozonation alone. Furthermore, loading transition metal oxides, such as MnO_2_ [16], CuO [17], ZnO, TiO_2_, and Fe_2_O_3_ [18]. on γ-Al_2_O_3_ markedly increases catalytic activity because transition metal oxides have easily accessible multiple oxidation states [19] that promote ozone decomposition. Manganese dioxide (MnO_2_) and copper oxide (CuO) particles have abundant surface basic sites [20] and have promising potential in degrading TROPs in coal chemical wastewater-biotreated effluent. Qi et al. synthesized MnO_2_-modified bauxite via wetness impregnation for use as a heterogeneous catalyst to ozonate 2,4,6-trichloroanisole (TCA) in water. The results indicated 85.02% TCA removal at pH 6.5 and is 1.5 times higher than that with ozonation alone [21]. The surface of cerium oxide (CeO_2_) forms rich Lewis acid sites and -OH groups, which are the initiators of ozone decomposition to generate ·OH. Therefore, CeO_2_-aided heterogeneous catalytic ozonation results in the mineralization of TROPs. Zhang et al. synthesized a CuO/CeO_2_ composite through impregnation and used it to catalyze atrazine ozonation. With CuO/CeO_2_ as a catalyst, 60% degradation efficiency was obtained at pH 6.7; meanwhile, ozonation alone has a degradation efficiency of 20% [22]. Rui et al. prepared a Mn-Ce-O catalyst by using the co-precipitation method. The use of the catalyst enhanced total phenolic content (TPh) and TOC degradation. Single ozonation achieved 88% and 24% of reduction for TPh and TOC after 120 min of reaction, respectively, whereas, catalytic ozonation with the catalyst load optimized (10 g/L) leads to total degradation of the phenolic content in 40 min and a final TOC reduction up to 74% [23]. Experimental results usually indicate that the removal efficiencies of pollutants are significantly enhanced in the presence of catalysts compared with that of ozone alone, and the multiple metal oxide-loaded γ-Al_2_O_3_ shows higher catalytic activity than that of single metal oxide loaded γ-Al_2_O_3_ due to the synergistic effects of multiple metal oxides [24]. To our best knowledge, no study has been conducted on heterogeneous catalytic ozonation with Cu-Mn-Ce@γ-Al_2_O_3_ as a multiple metal oxide-loaded catalyst in the advanced treatment of real biologically pretreated coal chemical wastewater.

This work aimed to fabricate a novel catalyst Cu-Mn-Ce@γ-Al_2_O_3_ by using a simple impregnation—calcination method—and to investigate its performance and the mechanism of catalytic ozonation. Scanning electron microscopy (SEM), energy dispersive spectrometry (EDS), X-ray diffraction (XRD), and Brunauer-Emmett-Teller (BET) analysis were used in catalyst characterization. The effects of the operating parameters on the catalytic ozonation in coal chemical wastewater-biotreated effluent were determined. The catalytic mechanism was revealed through GC-MS analysis, UV-visible spectroscopy, and FT-IR spectrometer analysis. The reusability and stability of the catalyst were evaluated.

## 2. Materials and Methods

### 2.1. Materials

Mn(NO_3_)_2_, Cu(NO_3_)_2_, Ce(NO_3_)_2_, and γ-Al_2_O_3_ were obtained from Aladdin Reagent (Shanghai, China). All other reagents were of analytical grade from Sinopharm Chemical Reagent Co., Ltd (Shanghai, China). The wastewater samples were SBR effluent collected from a coal chemical plant. The characteristics of industrial wastewater are shown in Table 1. The parameter of UV_254_ was directly related to the content of compounds with aromatic structures or unsaturated double bonds [25]. UV_410_ was used to indirectly characterize the chromaticity of wastewater [26]. The concentration of volatile phenol was determined by 4-APP spectrophotometry (HJ 503-2009).

### 2.2. Cu-Mn-Ce@γ-Al_2_O_3_ Preparation

Metal oxides were loaded onto γ-Al_2_O_3_ by incipient wetness impregnation. In brief, 5 g of γ-Al_2_O_3_ was first poured onto 40- and 60-mesh sieves to obtain identifiable particles. After particle sieving, the samples were washed for several times with deionized water and dried at 378 K for 13 h, followed by calcination at 673 K for 5 h. Then, pretreated γ-Al_2_O_3_ was dipped into 0.01 mol/L Ce(NO_3_)_2_ solution for 13 h. After filtration, the wet samples were dried at 393 K for 12 h and calcinated using a muffle furnace at a set temperature for several hours. Next, this catalyst was impregnated by 0.05 mol/L Cu(NO_3_)_2_ solution and 0.05 mol/L Mn(NO_3_)_2_ solution in turn, and the above procedures were repeated to obtain the Cu-Mn-Ce@γ-Al_2_O_3_ catalyst.

### 2.3. Cu-Mn-Ce@γ-Al_2_O_3_ Characterization

Elemental analysis was conducted using scanning electron microscope (SEM) equipped with energy dispersive X-ray spectroscopy (SEM-EDS, S-3400N II, Hitachi, Marunouchi, Japan) at 20 kV. XRD spectra were recorded on a diffractometer with Cu Kα source (X’TRA, ARL, Ecublens, Switzerland) The surface and pore size distribution of the prepared catalyst were characterized using N_2_ adsorption-desorption isotherms at 77 K with a surface and pore analyzer (Nova 3000, Quantachrome, Boynton Beach, FL, USA).

### 2.4. Catalytic Ozonation Procedure

**Figure 1:**
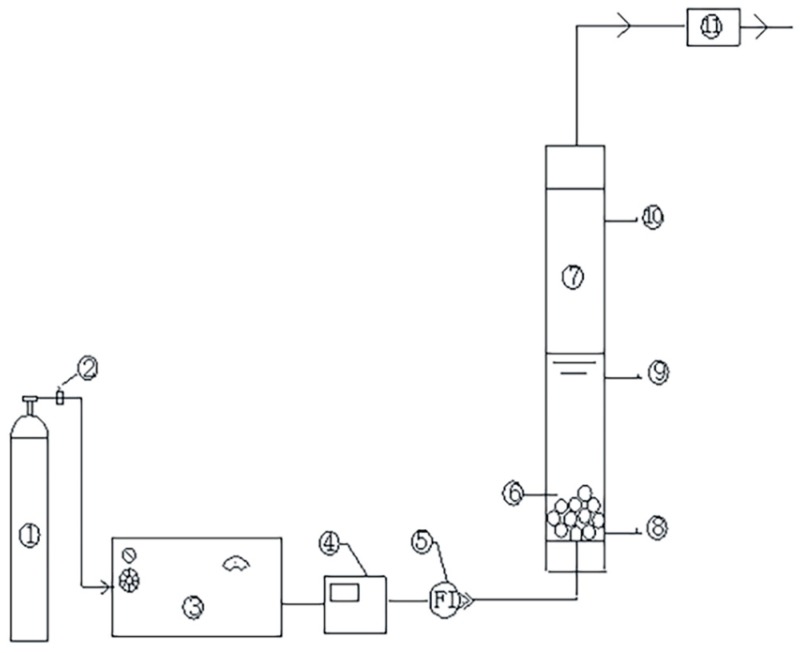
Schematic diagram of the laboratory ozonation system. (**1**) Oxygen tank. (**2**) Pressure reducing valve. (**3**) Ozone generator. (**4**) Ozone concentration detector. (**5**) Flow meter. (**6**) Catalyst. (**7**) Catalytic reactor. (**8**) Vent port. (**9**) Water sample ports. (**10**) Water inlet. (**11**) Exhaust gas absorption device.

The catalytic ozonation system consisted of an oxygen cylinder, odor ozone concentration detector, ozone generator, catalytic reactor, and exhaust gas absorption device (Figure 1). A catalytic reactor was constructed from polymethyl methacrylate (PMMA). The reactor had a cylinder shape with a diameter of 4 cm and a length of 22 cm. Ozone was generated in dry O_2_ by an ozone generator (COM-AD-01, ANSEROS, Tubingen, Germany) for the experiments. Different dosages can be obtained by adjusting the power of the ozone generator to control the outlet O_3_ concentration. 

Before each test, fresh catalyst was loaded into the reaction column. The coal chemical wastewater-biotreated effluent entered from the top of the reaction column and flowed out from the bottom. Ozonized oxygen was continuously bubbled into the reactor through a titanium microporous diffuser located at the reactor bottom. Excess ozone was treated and then discharged. Samples were collected from the sampling port at certain time intervals for advanced analysis [27,28]. All experiments were carried out at room temperature and atmospheric pressure.

### 2.5. Analytical Methods

Gas chromatographic-mass spectrometry (GC-MS) (7890AGC/597D, Agilent Technologies Co., Ltd, Santa Clara, CA, USA) was used to analyze the composition changes of organic pollutants before and after catalytic ozone oxidation. A UV-vis spectrophotometer (722N, Shanghai Precision Scientific Instruments Co., Ltd, Shanghai, China) (190–780 nm) and FT-IR spectrometer (IRAffinity-1, SHIMADZU Co., Ltd, Shanghai, China) (4000–400 cm^−1^) were used to analyze the structure and group changes of organic contaminants in wastewater before and after catalytic ozone oxidation. The concentration of ozone in aqueous solution was measured with an odor ozone concentration detector (PCII, Hach Company, Loveland, CO, USA). TOC was determined with a TOC analyzer (TOC-LCPH, SHIMADZU Co., Ltd, Shanghai, China). COD was measured via the potassium dichromate method (GB 11914-89, China). UV_254_ and UV_410_ were determined via spectrophotometry.

## 3. Results and Discussion

### 3.1. Adsorption Performances of Cu-Mn-Ce@γ-Al_2_O_3_

Alumina was used as a carrier for the catalyst. Alumina has a large specific surface area and can effectively adsorb organic pollutants in water [29]. Therefore, adsorption experiments were carried out immediately to evaluate the main function of Cu-Mn-Ce@γ-Al_2_O_3_ in TROP degradation. The catalysts contributed to the removal of COD, UV_254_, and UV_410_ by 11.1%, 3.3%, and 1.4%, respectively (Table 2).

The result suggested that the adsorption performance of Cu-Mn-Ce@γ-Al_2_O_3_ in the degradation of coal chemical wastewater-biotreated effluent was low because TROPs in biotreated effluent were mainly in dissolved state after physicochemical pretreatment and biochemical secondary treatment limited the adsorption performance on Cu-Mn-Ce@γ-Al_2_O_3_ [30]. 

### 3.2. Optimization of Catalyst Preparation Conditions

#### 3.2.1. Effects of Metal Oxide-Loading Percentage

Proper catalyst composition is crucial to the performance because insufficient metal oxide loading percentage may cause lack of active sites, but in excess, clusters of metal oxides might form on the catalyst surface, resulting in decreased catalytic activity [31]. Therefore, percentages of metal oxides loaded onto γ-Al_2_O_3_ were first optimized for TOC removal.

The optimization experiments were based on an orthogonal array experiment design, and the following three variables were analyzed on the basis of metal oxide (i.e., MnO_2_, CuO, and CeO_2_) loading percentage. These three factors have significant effects on the catalytic ozonation of Cu-Mn-Ce@γ-Al_2_O_3_. Above all, an L9 (3^3^) matrix is an orthogonal array of three factors and three levels (Table 3). A series of catalysts with different ratios of metal oxide loading percentages were obtained by altering the concentration of the metal nitrate solution (Table 3) [32,33].

The orthogonal experiment results are listed in Table 4. All the experiments were aimed at increasing the removal rate of COD. R indicated the significance of a factor and a larger R meant that this factor had a more significant impact on the removal rate. Based on the comparison of R, the prominence order of the metal oxides loading rate was: CuO (19) > CeO_2_ (7.93) > MnO_2_ (2.73). It can be seen from the k value in Table 4 that the optimum reaction conditions for the catalyst were that the loading mass fractions (ω) of CuO, MnO_2_, and CeO_2_ were 17%, 3%, and 2%, respectively. According to this condition, the removal rate of the average COD of the coal chemical wastewater-biotreated effluent was 61%. The results of the five verification experiments are shown in Table 5.

#### 3.2.2. Effects of Calcination Temperature

**Figure 2:**
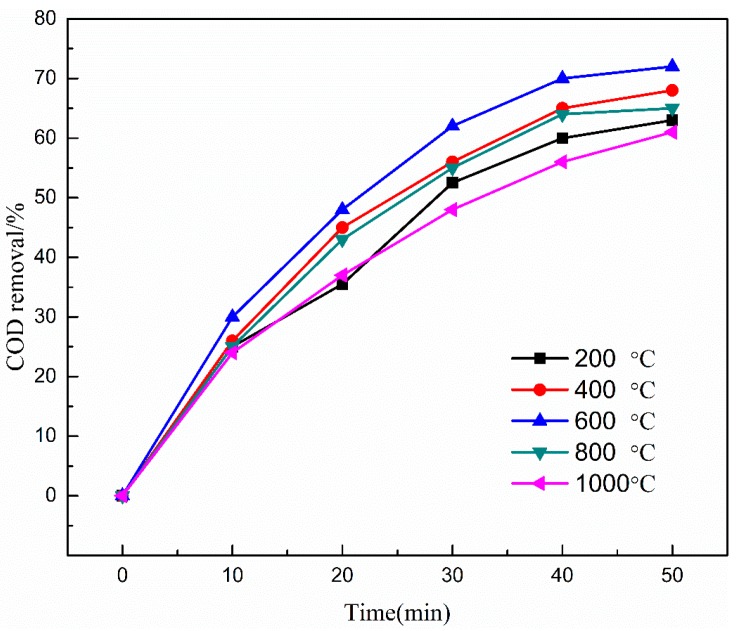
Effect of calcination temperature on COD removal efficiency. Catalyst preparation conditions: the optimal loading ratio of metal oxides; calcination time, 5 h; ozone dosage, 80.0 mg/L; initial solution pH, 7; reaction time, 50 min.

**Figure 3:**
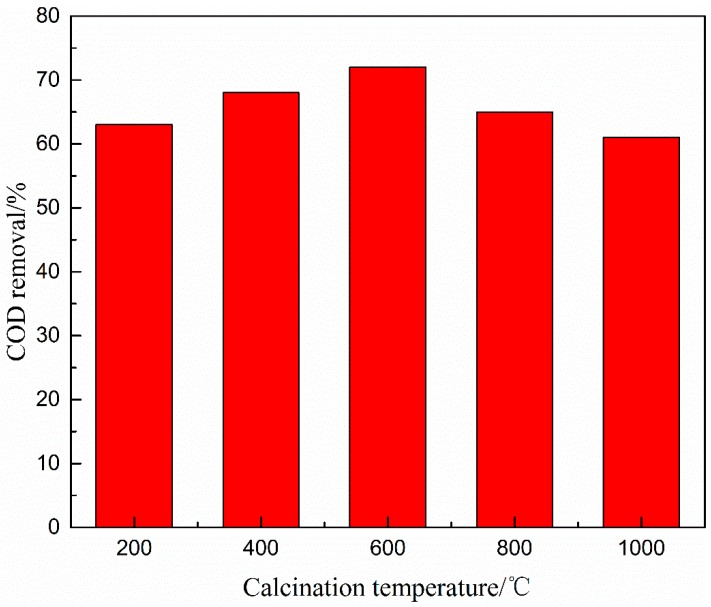
COD removal versus calcination temperature at reaction time of 50 min.

The efficiencies of COD removal from coal chemical wastewater biotreated effluent were evaluated by using catalysts prepared at various calcination temperatures (i.e., 200 °C, 400 °C, 600 °C, 800 °C, and 1000 °C). As illustrated in Figure 2 and Figure 3, the COD removal efficiency increased initially and then declined with increasing temperature and reached the uppermost value at 600 °C calcination temperature. 

Thus, low calcination temperatures lead to insufficient active sites, but high temperatures cause active sites to agglomerate [34]. This phenomenon could be attributed to the following factors: (i) As calcination temperature increased, the catalyst surface of active sites increased at the calcination temperature below 600 °C but decreased above 600 °C, thereby limiting the catalytic activity of Cu-Mn-Ce@γ-Al_2_O_3_. (ii) The mechanical strength could be enhanced with increased calcination temperature. However, the catalyst channels would break at very high temperatures (>600 °C). Thus, suitable mechanical strength and the catalyst channels of Cu-Mn-Ce@γ-Al_2_O_3_ could improve the catalytic performance [35]. Therefore, considering the COD removal efficiency and catalyst stability, 600 °C was considered and applied as the suitable calcination temperature throughout the following experiment. In addition, it can be found that the reaction time also had a significant effect on the COD removal efficiency from the above experiments and the effect of reaction time by catalytic ozonation is given in Section 3.4.3.

#### 3.2.3. Effects of Calcination Time

**Figure 4:**
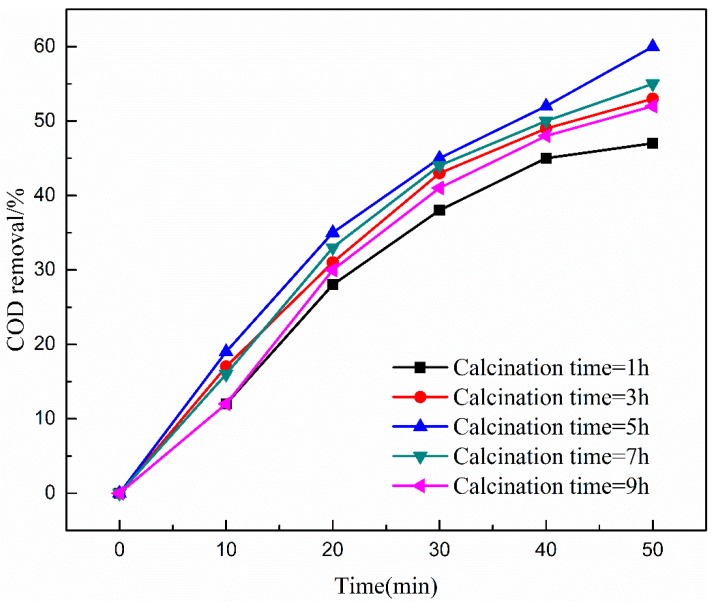
Effect of calcination time on COD removal efficiency. Catalyst preparation conditions: optimal loading ratio of metal oxides; calcination temperature, 873 K; ozone dosage, 80.0 mg/L; initial solution pH, 7; reaction time, 50 min.

**Figure 5:**
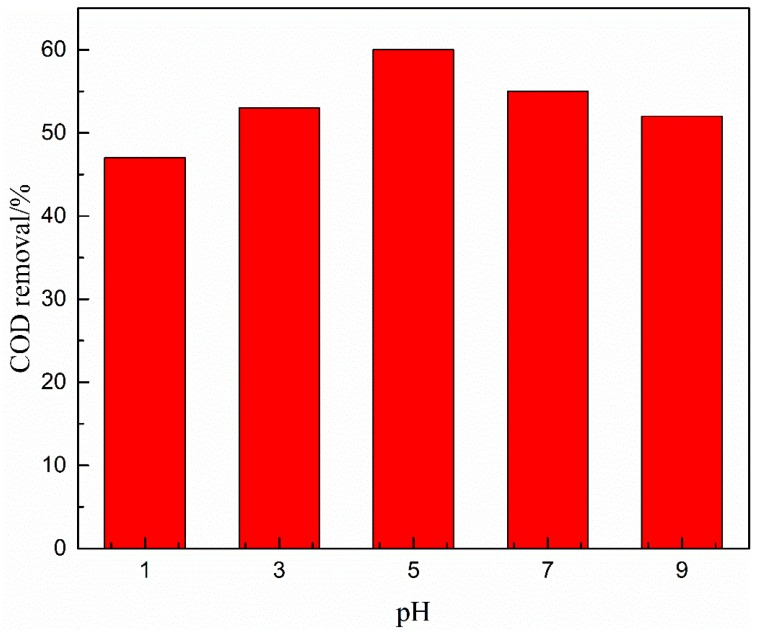
COD removal versus calcination temperature at reaction time of 50 min.

The effects of catalysts prepared at various calcination times (i.e., 1, 3, 5, 7, and 9 h) on COD removal efficiency from coal chemical wastewater-biotreated effluent was investigated. As shown in Figure 4 and Figure 5, calcination time from 1 to 5 h improved the COD removal efficiency after 50 min catalytic ozonation with Cu-Mn-Ce@γ-Al_2_O_3_, while the COD removal efficiency decreased with calcination time of 5 to 9 h. The catalytic activity of Cu-Mn-Ce@γ-Al_2_O_3_ reached peak value through 5 h calcination time and the COD removal efficiency was 61%.

The results can be attributed to the following reasons [36]: (i) The transition metal (i.e., Cu, Mn, Ce) could not be completely oxidized to generate high active components in the surface through shorter calcination time (<5 h). (ii) Cu-Mn-Ce@γ-Al_2_O_3_ formed a suitable catalytic surface, active sites, crystal form, and crystallite size when calcination time reached up to 5 h. (iii) However, if the calcination time is too long, metal oxides would sinter together, which can decrease the specific surface area and the dispersion of metal oxides. Considering the cost, time, and the COD removal efficiency, 5 h calcination was selected as an optimal calcination time in the experiments.

### 3.3. Characterization of Catalysts

#### 3.3.1. Scanning Electron Microscopy (SEM) and Energy Dispersive Spectrometer (EDS)

**Figure 6:**
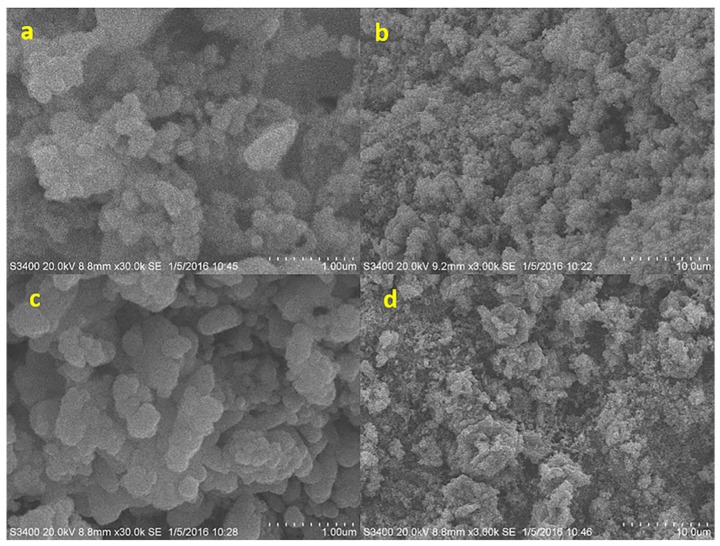
Scanning Electron Microscopy (SEM) images: (**a**) and (**b**) Cu-Mn-Ce@γ-Al_2_O_3_; (**c**) γ-Al_2_O_3_; (**d**) Cu-Mn@γ-Al_2_O_3_.

In Figure 6, the surface of the catalyst support was formed by crystal grains and fine pores, and the surface pores of Cu-Mn-Ce@γ-Al_2_O_3_ were significantly increased. Furthermore, the particle size of Cu-Mn-Ce@γ-Al_2_O_3_ catalyst was obviously smaller and more uniform than that of γ-Al_2_O_3_. However, several small and non-obvious crystal grains were formed on the surface of the catalyst support.

Cu-Mn-Ce@γ-Al_2_O_3_ catalyst loaded with metal oxides was highly likely to adsorb organic pollutants, resulting in increased conduciveness to ozone decomposition on its surface and increased catalytic activity [37]. Above all, the metal oxides (CuO-MnO_2_-CeO_2_) were deposited on the γ-Al_2_O_3_ surface to form micro-agglomerates in irregular shapes and sizes (Figure 6), which promoted interactions of Cu-Mn-Ce@γ-Al_2_O_3_ and TROPs in coal chemical wastewater-biotreated effluent. As shown in Figure 6b,d, the surface dispersibility of the catalyst was obviously improved after cerium oxide was added. The pore diameter was uniform, and the active center crystal grains were smaller. Cerium oxide was beneficial in increasing the catalytic activity of the catalyst.

**Figure 7:**
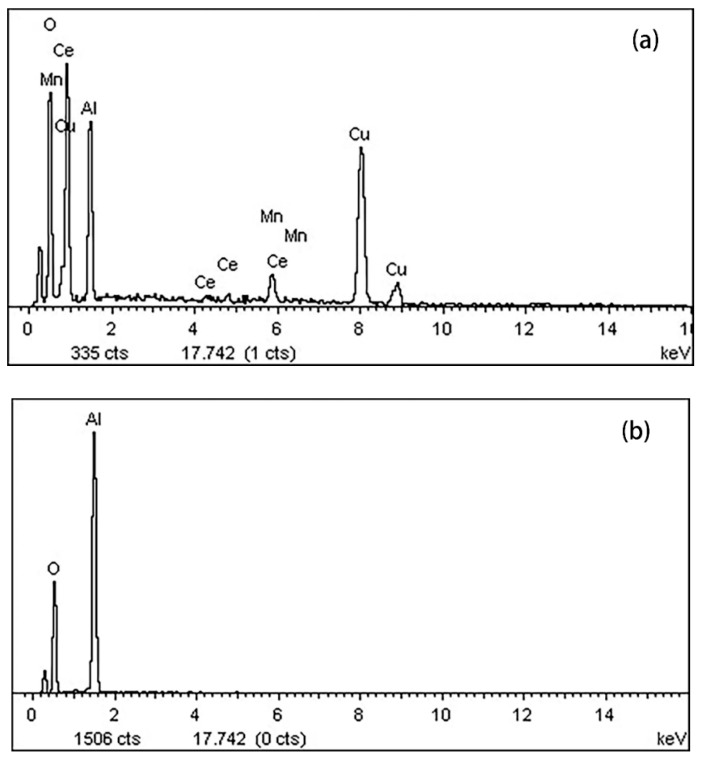
Surface atoms: (**a**) Cu-Mn-Ce@γ-Al_2_O_3_; (**b**) γ-Al_2_O_3_.

EDS mapping of Cu-Mn-Ce@γ-Al_2_O_3_ prepared via impregnation–calcination and γ-Al_2_O_3_ is shown in Figure 7a,b. Cu, Mn, Al, and Ce were distributed on the surface of Cu-Mn-Ce@γ-Al_2_O_3_, but only Al and O were observed on γ-Al_2_O_3_ and were similar to SEM images. The results indicated that Cu, Mn, and Ce were successfully loaded onto γ-Al_2_O_3_, and Cu-Mn-Ce@ γ-Al_2_O_3_ exhibited high-activity performance.

#### 3.3.2. X-ray Diffraction (XRD)

**Figure 8:**
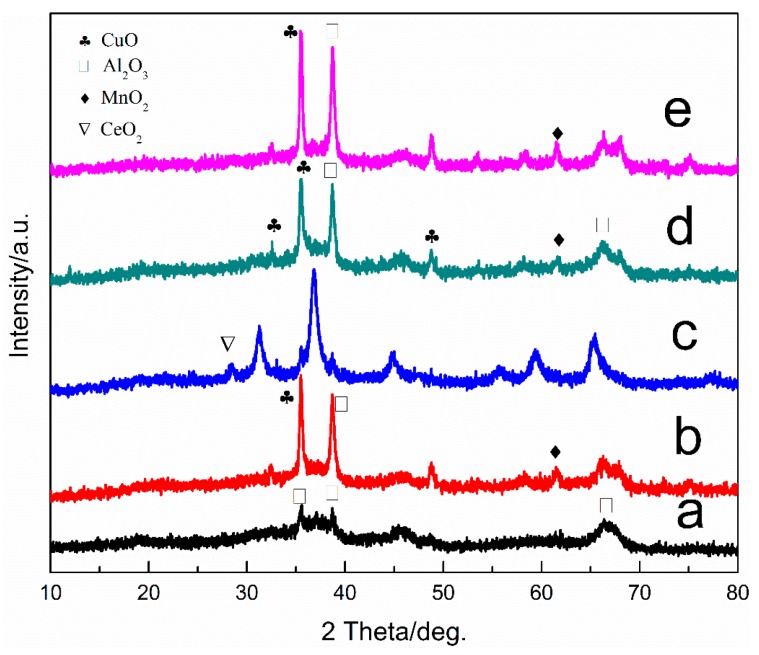
XRD patterns: (**a**) γ-Al_2_O_3_; (**b**) MnO_2_-CuO/γ-Al_2_O_3_; (**c**) Cu-Mn-Ce@γ-Al_2_O_3_ calcined at 1000 °C; (**d**) Cu-Mn-Ce@γ-Al_2_O_3_ calcined at 600 °C;(**e**) Cu-Mn-Ce@γ-Al_2_O_3_ calcined at 200 °C.

Cu-Mn-Ce@γ-Al_2_O_3_ catalyst showed typical XRD pattern peaks of MnO_2_, CuO, and γ-Al_2_O_3_ (Figure 8). The diffraction peak of CuO was sharp, indicating that the catalyst surface was highly crystalline. No obvious MnO_2_ XRD diffraction peak was observed, indicating that MnO_2_ dispersed well on the γ-Al_2_O_3_ surface. Compared with the diffraction peak of γ-Al_2_O_3_, some of the characteristic peaks of γ-Al_2_O_3_ disappeared in the catalyst diffraction peaks. This phenomenon may be due to the change of crystal structure caused by other metals that entered the lattice of γ-Al_2_O_3_ upon the preparation of the Cu-Mn-Ce@γ-Al_2_O_3_ catalyst.

To investigate the effect of Ce on the catalytic activity, the Cu-Mn-Ce@γ-Al_2_O_3_ catalyst and the Ce-free catalyst were characterized using XRD. As shown in Figure 8, after CeO_2_ modification was added, the degree of crystallization of the surface active component of the Cu-Mn-Ce@γ-Al_2_O_3_ catalyst was obviously increased, and the crystal grains became larger. Thus, CeO_2_ modification can effectively inhibit MnO_2_ and CuO from entering the carrier γ-Al_2_O_3_ lattice, promote the dispersion of the catalyst surface, and improve the catalyst activity.

Furthermore, the effect of calcination temperature on catalytic structures was characterized through XRD. As shown in Figure 8, the different peaks in XRD pattern were analyzed and compared with a JCPDS (Joint Committee on Powder Diffraction Standards) card, thereby obtaining the corresponding catalyst composition. The results showed that γ-Al_2_O_3_ and CuO crystal phases were on the surface of Cu-Mn-Ce@γ-Al_2_O_3_ prepared at 200 °C. The XRD diffraction peak broadening of MnO_2_ was relatively serious, indicating that the surface Mn component of the catalyst was mainly present in an amorphous form. 

At the calcination temperature of 600 °C, the diffraction peak intensity of CuO and MnO_2_ strengthened, and the crystalline diameter became slightly larger. At this temperature, the catalyst surface had numerous effective active sites, and the crystal grains were relatively dispersed, which improved the catalytic activity. However, continued increase in calcination temperature to 1000 °C resulted in the poor dispersion of the metal oxide crystal on the catalyst. A large grain size and sintering phenomenon also reduced catalytic activity. The crystallite size increased with calcination temperature, and the small crystallite size might have more active sites and better catalytic performance than larger ones. Therefore, CuO and MnO_2_ were the active components of Cu-Mn-Ce@γ-Al_2_O_3_.

#### 3.3.3. Brunauer-Emmett-Teller (BET) Analysis

**Figure 9:**
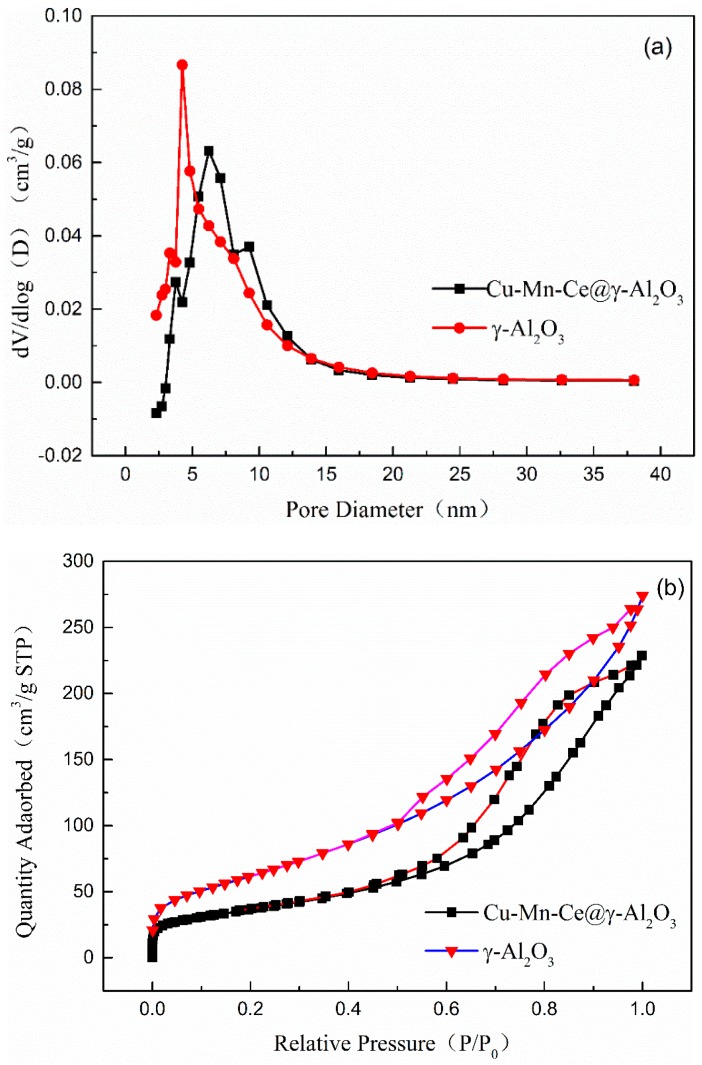
(**a**) Pore size distribution curves of the γ-Al_2_O_3_ and Cu-Mn-Ce@γ-Al_2_O_3_; (**b**) N_2_ adsorption-desorption isotherms.

BET analysis was performed on the catalyst carrier and the Cu-Mn-Ce@γ-Al_2_O_3_ catalyst (Table 6). Metal oxide loading on γ-Al_2_O_3_ decreased S_BET_ but increased the pore volumes (V_P_) and d_P_ from the pore distribution (Figure 9). 

The results can be explained as follows [38]: (i) The porous structure was blocked by metal oxides, leading to reduction in specific surface area. (ii) During high-temperature calcination of the Cu-Mn-Ce@γ-Al_2_O_3_ catalyst, the internal pores agglomerated. (iii) The average pore size of the catalyst increased, which might be due to the decreased number of pores after the metal oxides were loaded.

The nitrogen adsorption-desorption isotherms presented in Figure 6 illustrate the evolution of pore structure and quantity from the catalytic support to Cu-Mn-Ce@γ-Al_2_O_3_. The catalyst hysteresis loop was closed late. On the basis of the capillary condensation theory, the adsorption of narrow mesopores was initially carried out, and the wider mesopores were desorbed initially during desorption. Therefore, the number of mesopores on the surface of the catalyst was increased after calcination.

### 3.4. Effects of Operational Parameters

#### 3.4.1. Effects of Ozone Dosage

**Figure 10:**
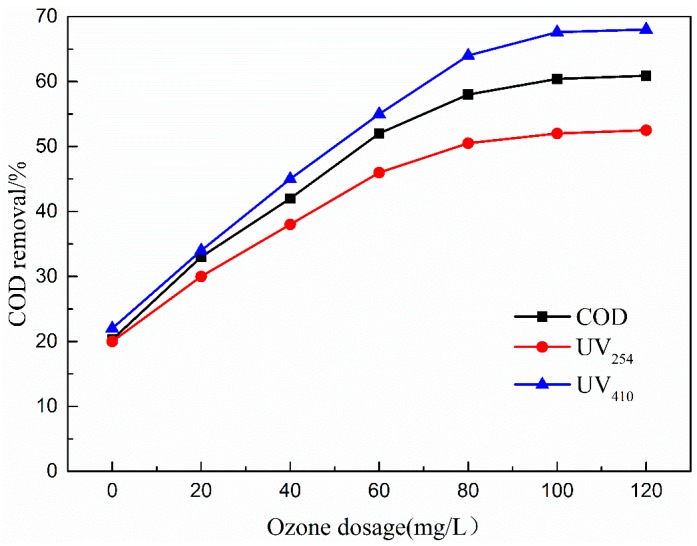
Effect of ozone dosage on COD removal efficiency. Catalytic ozonation operational parameters: wastewater sample, 1 L; reaction temperature, 293 K; catalyst dosage, 20.0 mg/L; initial solution pH, 7; reaction time, 50 min.

Ozone is a powerful oxidizing agent, and its dosage is determined by two factors, namely, flowrate and gas concentration [39]. In the current study, the effects of ozone gas concentration on COD, UV_254_, and UV_410_ removal efficiency were analyzed. COD removal efficiency increased gradually with increasing ozone dosage, and approximately 61% of COD was removed via catalytic ozonation to the ozone dosage of 80.0 mg/L (Figure 10). Catalytic activity gradually became stable with further increase in ozone dosage.

The rate of increase of removal efficiency gradually slowed down during the increase of ozone dosage from 80 mg/L to 120 mg/L because the concentration of organic pollutants in the wastewater gradually decreased with the increase of ozone dosage by catalytic ozonation and the remaining organic pollutants were structurally stable. Therefore, the existing catalytic ozonation system was difficult to cause further mineralization. Thus, the optimal ozone dosage of 80.0 mg/L was selected as a beneficial operation parameter in the following experiments.

#### 3.4.2. Effects of Catalyst Dosage

**Figure 11:**
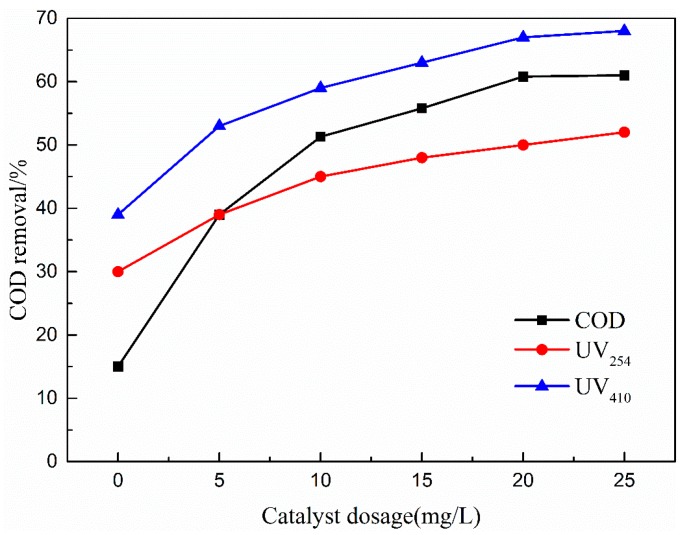
Effect of catalyst dosage on COD removal efficiency. Catalytic ozonation operational parameters: wastewater sample, 1L; reaction temperature, 293 K; ozone dosage, 80.0 mg/L; initial solution pH, 7; reaction time, 50 min.

Figure 11 presents the influence of catalyst dosages on COD, UV_254_, and UV_410_ removal efficiency. As shown in Figure 11, the low COD removal of 30% was obtained after 50 min of treatment by ozone alone. COD removal efficiency rapidly increased to 36% with 5 mg/L catalyst addition. The COD removal gradually increased to 61% as the Cu-Mn-Ce@γ-Al_2_O_3_ catalyst concentration increased to 20 mg/L. However, COD removal declined upon increasing catalyst dosage to 25 mg/L.

This phenomenon may be attributed to ·OH quenching. With increased catalyst dosage at a suitable range, Cu-Mn-Ce@γ-Al_2_O_3_ had an increased number of catalytic active sites. Thus, the reaction between coal chemical wastewater biotreated effluent and Cu-Mn-Ce@γ-Al_2_O_3_ was enhanced, and ozone decomposition was promoted to generate ·OH. However, a part of excess ·OH was quenched, which limited the degradation of coal chemical wastewater-biotreated effluent and decreased COD removals [40]. Thus, 20 mg/L was selected as the optimal catalyst dosage in the current study.

#### 3.4.3. Effects of Reaction Time

**Figure 12:**
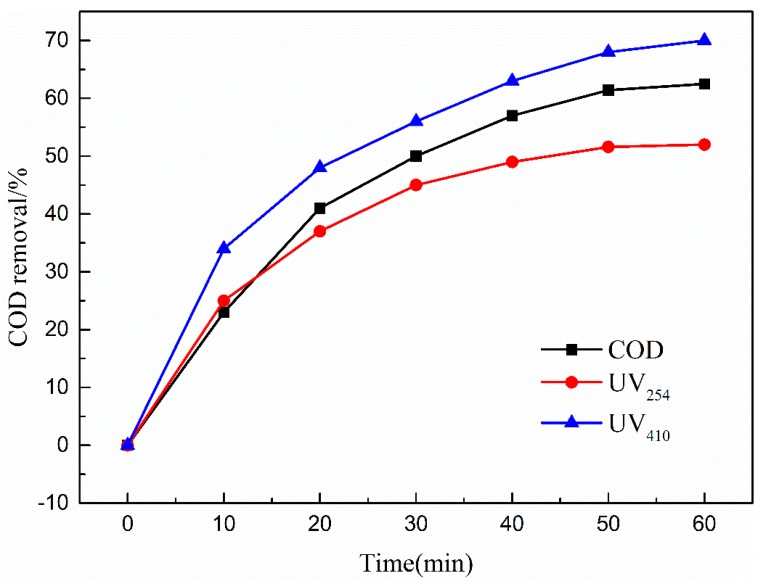
Effect of reaction time on COD removal efficiency. Catalytic ozonation operational parameters: wastewater sample, 1 L; reaction temperature, 293 K; ozone dosage, 80.0 mg/L; catalyst dosage, 20.0 mg/L; initial solution pH, 7.

Figure 12 presents the effect of reaction time on COD, UV_254_, and UV_410_ removal efficiencies. As shown in Figure 12, the removal rates of COD, UV_254_, and UV_410_ in wastewater also increased with increasing reaction time. However, COD removal efficiency increased until the 50 min reaction time.

This phenomenon was due to the high initial concentration of organic matter in wastewater, and the ozone directly oxidized wastewater to be easily degraded at the beginning of the reaction. As the reaction proceeded, the concentration of organic pollutants gradually decreased, and the amount of organic matter that was difficult to degrade increased. The removal rate came to a plateau. Thus, the optimal reaction time of 50 min was selected to carry out the experiments.

#### 3.4.4. Effects of pH

**Figure 13:**
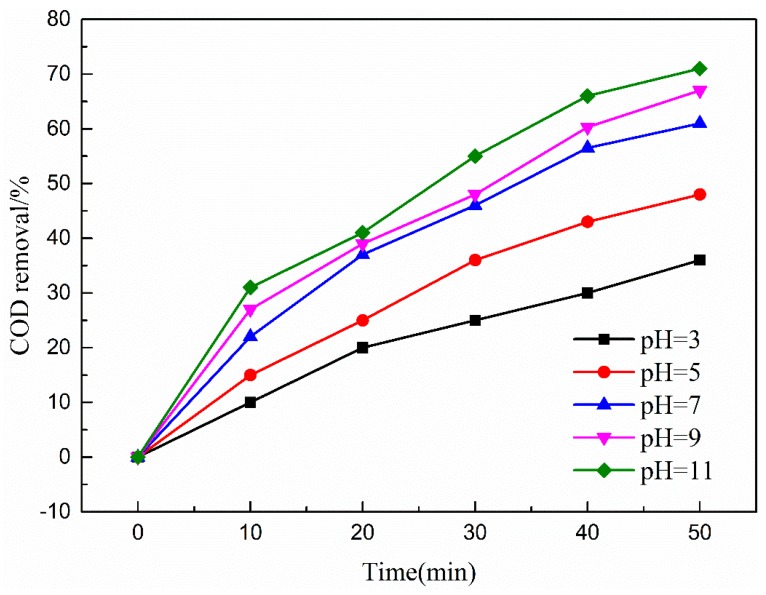
Effect of pH on COD removal efficiency. Catalytic ozonation operational parameters: wastewater sample, 1 L; reaction temperature, 293 K; ozone dosage, 80.0 mg/L; catalyst dosage, 20.0 mg/L; reaction time, 50 min.

The pH value of the solution may influence ozone decomposition and the surface characteristics of catalyst in catalytic ozonation. Figure 13 shows that the efficiency for coal chemical wastewater-biotreated effluent degradation in the O_3_- Cu-Mn-Ce@γ-Al_2_O_3_ catalytic ozonation system increased with initial pH values. COD removal efficiency in biotreated effluent attained 67% at initial pH of 9 at 50 min reaction time. 

The ·OH was easily formed by ozone decomposition in alkaline conditions and reacted with organics in a non-selective mode involving highly oxidative hydroxyl radical. However, in acidic conditions, ozone molecules selectively attack pollutants containing aromatic structures or unsaturated double bonds via direct ozonation, and ketones and lipids were produced in the effluent.

However, COD removal efficiency decreased at the continued increase of the pH value to 11, probably owing to the increase of OH- in wastewater that reduced the concentration of hydroxyl radicals.

Furthermore, this result might indicate that pH could also have a great influence on the surface properties of Cu-Mn-Ce@γ-Al_2_O_3_; pH_PZC_ is the pH of the zero charge point of the catalyst surface. At aqueous solution pH near the pH_PZC_ of the catalyst, most of the surface hydroxyl radicals are in a neutral state, where the surface ·OH achieves a relatively high catalytic activity to improve the ozone decomposition and ·OH generation. However, at pH above pH_PZC_, the catalyst surface becomes protonated or deprotonated, and catalyst activity and treatment efficiency of coal chemical wastewater biotreated effluent decreased [41]. The initial pH of 9 was selected as the optimal operational pH value in the subsequent experiment.

### 3.5. Mechanisms of Heterogeneous Catalytic Ozonation

#### 3.5.1. GC-MS Analysis

**Figure 14:**
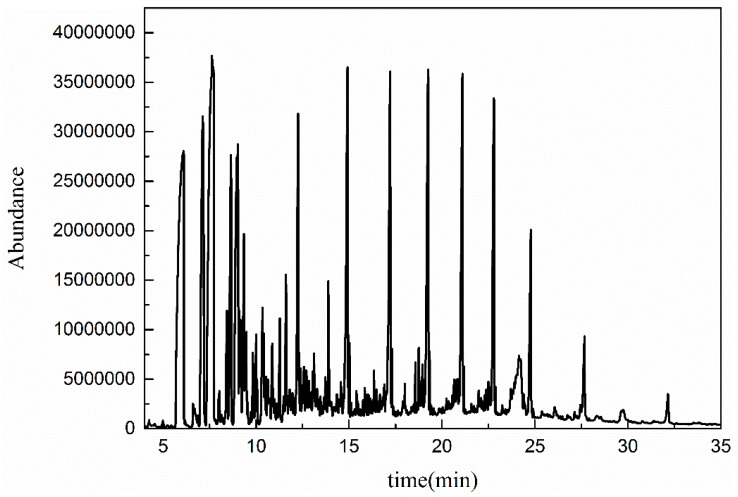
Organic compounds before catalytic oxidation.

**Figure 15:**
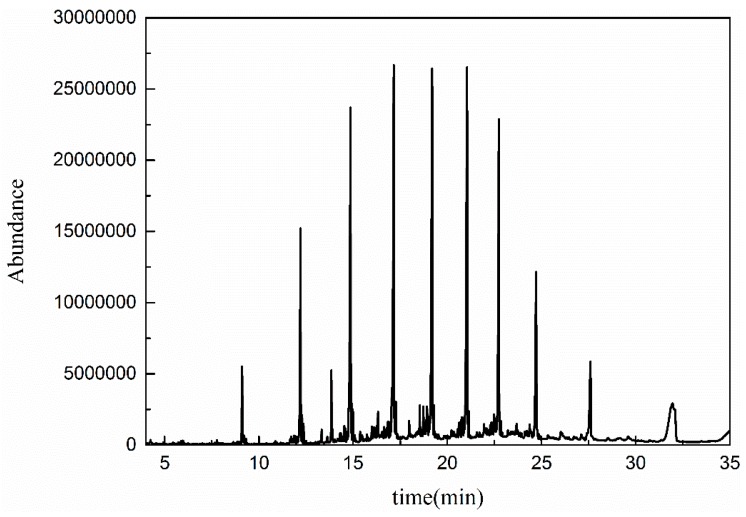
Organic compounds after catalytic oxidation.

To further demonstrate the mechanisms of heterogeneous catalytic ozonation, the molecular weight distribution of organic contaminants in wastewater before and after catalytic ozonation was determined using GC-MS. As shown in Figure 14 and Figure 15, the spectra of the coal chemical wastewater biotreated effluent before and after treatment showed similar peaks with different intensities [42]. The polycyclic aromatic hydrocarbons and nitrogenous heterocyclic compounds decreased after catalytic ozonation. The main hydrocarbons in treated effluent were alcohols and lipids (Table 7).

The GC-MS results indicated that the contaminants with heterocyclic structures and polycyclic aromatic hydrocarbons in coal chemical wastewater-biotreated effluent were degraded by ·OH in the catalytic ozonation system. This process generated olefins and alcohols with simple structures. Thus, catalytic ozonation was more efficient than ozonation in degrading polycyclic aromatic hydrocarbons and nitrogenous heterocyclic compounds. Long-chain alkanes, macromolecular lipids, and pollutants with symmetrical structures were not completely mineralized via catalytic ozonation.

#### 3.5.2. UV-vis Spectroscopy

**Figure 16:**
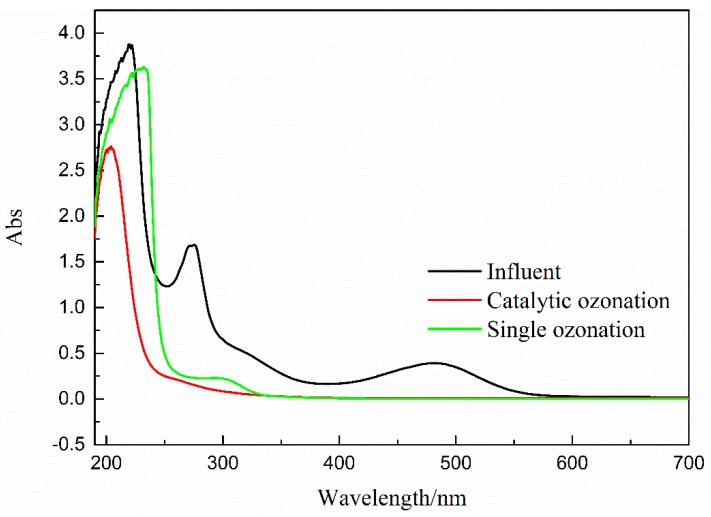
Changes in UV-vis spectra before and after catalytic ozonation.

As shown in Figure 16, the absorbance decreased with reaction time and indicated that the TROPs were partially degraded by catalytic ozonation in coal chemical wastewater biotreated effluent. Within the visible light range of 400–780 nm, the spectral absorption intensity of the effluents of catalytic ozonation and ozonation was almost zero, indicating the low chromaticity of the wastewater. The intensity of UV-absorption spectrum of the catalytic ozonation effluent decreased in each wavelength range (200–400 nm) and was obviously lower than that of ozonation, suggesting that catalytic ozone oxidation can effectively degrade the organic matter in wastewater. 

Furthermore, during catalytic ozonation, ozone attacked organic groups with wavelengths ranging from 220 nm to 295 nm. Thus, polycyclic aromatic hydrocarbons existed in biotreated effluent. After catalytic ozonation, a moderate absorption was observed in the 220–240 nm phase, and the strength was significantly reduced. These observations were consistent with GC-MS results (Figure 14 and Figure 15) and indicated that water contained double bond structures.

COD removal efficiency in catalytic ozonation was more than that in ozonation alone, proving that the utilization of ozone was enhanced in the presence of catalyst, owing to the production of a few reactive oxygen (·OH) [43].

#### 3.5.3. FT-IR Spectrometer Analysis

**Figure 17:**
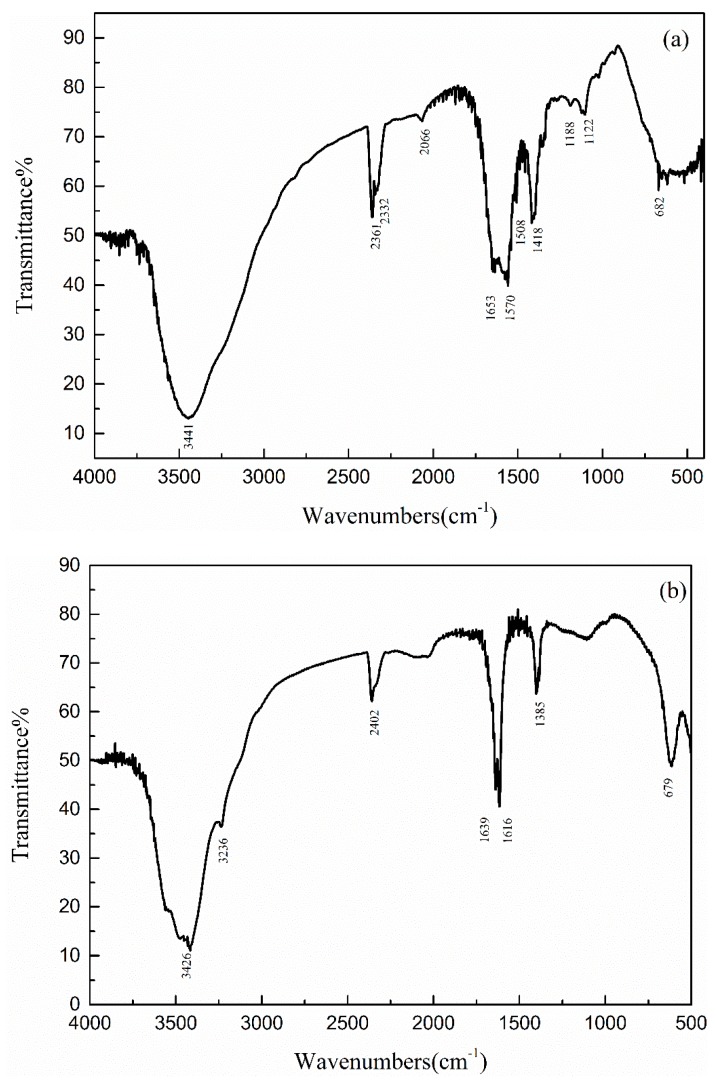
Changes in FT-IR spectra before and after catalytic ozonation: (**a**) Influent; (**b**) effluent.

FT-IR analysis of organic matter in wastewater was carried out before and after catalytic ozonation by using tableting method, and the results are shown in Figure 17. The structure and group information of organic pollutants in the wastewater before and after catalytic ozonation are shown in Table 8 and Table 9, respectively. After catalytic ozonation, -NO_2_ (aromatic), O-H deformation vibration, C=O stretching vibration, C-O-C stretching vibration, C-O stretching vibration, and C-N stretching vibration groups disappeared. The C=C bond of the aromatic ring was opened and became a small molecule olefin C=C bond. The ether C-O-C bond was oxidized to an O-H bond or -CH_3_, and the C-N bond became an N-H bond. 

Compared with that of ozonation alone, catalytic ozonation resulted in the disappearance of ether groups. The oxygen-containing functional groups increased, indicating that Cu-Mn-Ce@γ-Al_2_O_3_ can increase the oxidation potential of ozone and make the organic pollutants highly oxidized [44].

### 3.6. Stability of CMC-A

**Figure 18:**
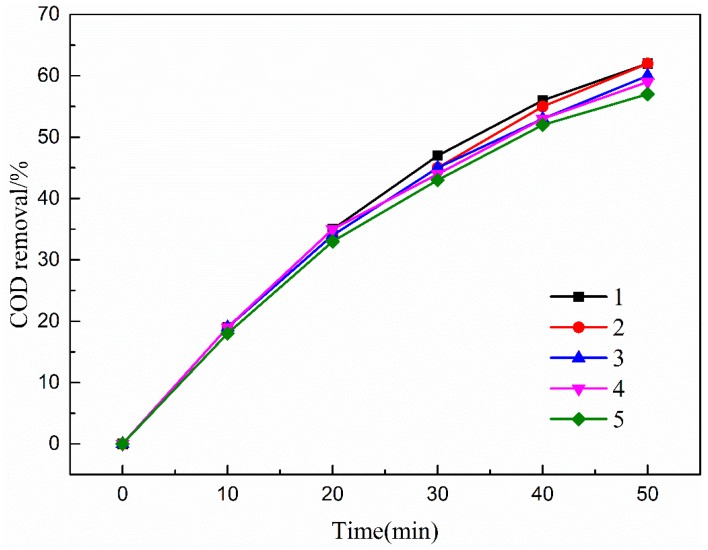
COD removal for five successive experiments. Experimental conditions: wastewater sample, 1 L; reaction temperature, 293 K; ozone dosage, 80.0 mg/L; initial solution pH, 7; reaction time, 50 min.

COD removal in five sequentially repeated catalytic ozonation experiments was monitored. COD removal efficiencies ranged from 57% to 61% (Figure 18). The excellent stability of the catalytic activity could be attributed to the stable structure of the catalyst.

**Figure 19:**
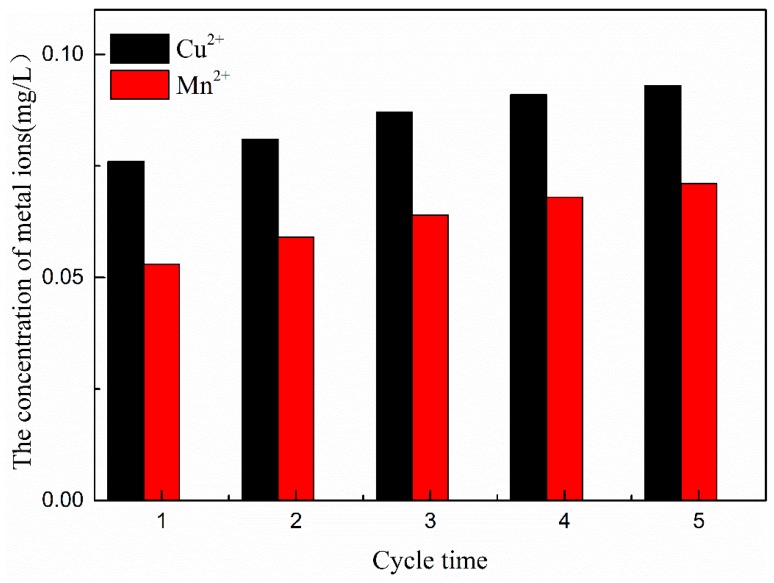
Cu-Mn-Ce@γ-Al_2_O_3_ catalyst metal dissolution test.

As shown in Figure 19, during catalytic ozonation, the concentrations of three metal ions of Cu, Mn, and Ce that dissolved in wastewater were negligible. After five experiments (i.e., 250 min in total), the metal Ce ions were not detected in the solution. The concentrations of Cu and Mn ions were 0.091 and 0.007 mg/L, which accounted for 0.0032% and 0.0018% of the total Cu and Mn in the catalyst, respectively. No evident metal elution from Cu-Mn-Ce@γ-Al_2_O_3_ was observed after five repeated uses (Figure 16). These results indicated that Cu-Mn-Ce@γ-Al_2_O_3_ was stable and could be repeatedly used.

## 4. Conclusions

The Cu-Mn-Ce@γ-Al_2_O_3_ prepared for catalytic ozonation in coal chemical wastewater-biotreated effluent was systematically investigated in this study. The following conclusions were drawn:

(1) The optimum metal oxide loading percentage of Cu-Mn-Ce@γ-Al_2_O_3_ are 17.0% (CuO), 3.0% (MnO_2_), and 2.0% (CeO_2_). The optimum calcination temperature is 600 °C and calcination time is 5 h for this catalyst.

(2) The surface of the catalyst was characterized using SEM-EDS, XRD, and BET techniques. CuO and MnO_2_ were the active components that improved the catalytic activity of Cu-Mn-Ce@γ-Al_2_O_3_. CeO_2_ enhanced the bonding strength between the active component and the catalyst support, thereby enhancing the stability of the catalyst.

(3) The optimal catalytic ozonation operating parameters, including ozone dosage, catalyst dosage, pH, and reaction time, were 80.0 mg/L, 20.0 mg/L, 7, and 50 min, respectively. The COD removal of biotreated effluent increased to 61% under these optimal operating conditions, compared with only 20% removal with ozonation alone.

(4) Degradation pathway analysis indicated that TROPs, such as phenolic compounds, polycyclic aromatic hydrocarbons, and NHCs, were degraded via catalytic ozonation to hydrocarbons containing a small amount of alcohols and lipids.

Therefore, this study provides a new insight into the application of catalytic ozonation in coal chemical wastewater biotreated effluent. The high removal efficiency and industrial feasibility suggest that heterogeneous catalytic ozonation is a promising technology for large-scale chemical wastewater treatment in the future.

## Figures and Tables

**Table 1:** Characteristics of the tested coal chemical biochemical tail water.

Parameter	COD (mg/L)	NH_3_-N (mg/L)	pH	UV_254_ (cm^−1^)	UV_410_ (cm^−1^)	Volatile Phenol (mg/L)
Average value	180.0	6.3	7.0–8.0	1.646	0.371	0.15

**Table 2:** Water quality before and after treatment.

Parameter	Influent	Effluent	Removal Efficiency (%)
COD (mg/L)	140.0	124.5	11.1
UV_254_	1.646	1.592	3.3
UV_410_	0.371	0.366	1.4

Operational parameters: wastewater sample, 1 L; reaction temperature, 293 K; catalyst dosage, 5 g; initial solution pH, 7; reaction time, 50 min.

**Table 3:** Levels and factors in orthogonal experiment design.

Level	Ω (CuO)/%	Ω (MnO_2_)/%	Ω(CeO_2_)/%
1	10.0	1.0	1.0
2	13.5	3.0	2.0
3	17.0	5.0	3.0

**Table 4:** Factorial design analysis experiments.

Trial Number	CuO	MnO_2_	CeO_2_	COD Removal Efficiency (%)
1	10	1	1	34.5
2	10	3	3	36.3
3	10	5	2	44.2
4	13.5	3	2	58.1
5	13.5	5	1	43
6	13.5	1	3	53
7	17	5	3	57
8	17	1	2	57
9	17	3	1	58
K_1_	115.02	144.51	135.51	/
K_2_	154.11	152.4	159.3	/
K_3_	172.02	144.21	146.38	/
k_1_	38.34	48.17	45.17	/
k_2_	51.37	50.8	53.1	/
k_3_	57.34	48.07	48.76	/
R	19	2.73	7.93	/

**Table 5:** COD removal efficiency after the test.

Trial Number	1	2	3	4	5
COD removal efficiency (%)	61.2	62.0	60.1	59.8	61.1

**Table 6:** BET analysis of catalyst.

Catalysts	S_BET_ (m^2^/g)	V_P_ (cm^2^/g)	d_P_ (nm)
γ-Al_2_O_3_	210	0.4192	4.25
Cu-Mn-Ce@γ-Al_2_O_3_	172.35	0.4304	7.1

**Table 7:** Organic compounds before and after the reaction in water composition.

Organic Matter.	Influent	Effluent
Hydrocarbon	tetradecene, hexadecene, nonadecane, heptadecene, octadecene, trihexene	Tetradecene, hexadecene, heptadecene, octadecene, dimethyl (dodecane)
Ketones	2-pentanone, cyclohexanone	-
Lipids	3-tetradecyl 3-fluorobenzoate, 2-ethyl-4-methylpentyl pentoxide, dibutyl phthalate	ethyl isothioate
Acids	Octanoic acid	-
Alcohols	2,3-dihydro-1H-indol-5-ol, behenyl alcohol, N-tetracosyl alcohol, octacosanol	1-tridecyl alcohol, octacosanol

**Table 8:** Organic Compounds of FT-IR before ozonation.

Absorption Peak	Functional Group
3441.01	N-H stretching vibration
1653.00	C=C stretching vibration, C=O stretching vibration
1570.06	N-H stretching vibration
1508.33	-NO_2_ (Aromatic)
1417.68	O-H deformation vibration
1188.15	C-O-C stretching vibration, C-O stretching vibration, C-N stretching vibration
682.30	C-H deformation vibration, N-H deformation vibration

**Table 9:** Organic Compounds of FT-IR after ozonation.

Absorption Peak	FUNCTIONAL GROUP
3425.93	N-H stretching vibration
3236.53, 2401.94	O-H stretching vibration
1639.49	C=C stretching vibration, C=O stretching vibration, N-H deformation vibration
1616.35	C=C stretching vibration, N-H deformation vibration
1384.89	-CH_3_ deformation vibration
679.29	C-H deformation vibration, N-H deformation vibration
